# Conflict in Protected Areas: Who Says Co-Management Does Not Work?

**DOI:** 10.1371/journal.pone.0144943

**Published:** 2015-12-29

**Authors:** Kobe De Pourcq, Evert Thomas, Bas Arts, An Vranckx, Tomas Léon-Sicard, Patrick Van Damme

**Affiliations:** 1 Laboratory of Tropical and Subtropical Agriculture and Ethnobotany, Ghent University, Gent, Belgium; 2 Conflict and Development studies, Ghent University, Gent, Belgium; 3 Instituto de Estudios Ambientales, Universidad Nacional de Colombia, Bogotá D.C., Colombia; 4 Bioversity International, regional office for the Americas, Cali, Colombia; 5 Forest and Nature Conservation Policy Group, Wageningen University, Wageningen, the Netherlands; 6 Faculty of Tropical AgriSciences, Czech University of Life Sciences Prague, Prague, Czech Republic; Swedish University of Agricultural Sciences, SWEDEN

## Abstract

Natural resource-related conflicts can be extremely destructive and undermine environmental protection. Since the 1990s co-management schemes, whereby the management of resources is shared by public and/or private sector stakeholders, have been a main strategy for reducing these conflicts worldwide. Despite initial high hopes, in recent years co-management has been perceived as falling short of expectations. However, systematic assessments of its role in conflict prevention or mitigation are non-existent. Interviews with 584 residents from ten protected areas in Colombia revealed that co-management can be successful in reducing conflict at grassroots level, as long as some critical enabling conditions, such as effective participation in the co-management process, are fulfilled not only on paper but also by praxis. We hope these findings will re-incentivize global efforts to make co-management work in protected areas and other common pool resource contexts, such as fisheries, agriculture, forestry and water management.

## Introduction

Protected areas are pivotal for preventing biodiversity loss worldwide [1,2]. Recent evidence suggests that protected areas inhabited and/or managed by traditional communities are generally better preserved than areas governed by exclusionary conservation policies [3,4]. This does not imply that such areas are devoid of problems. Their management generally involves continuous dialogue and negotiation between indigenous and local communities on the one hand and state and park authorities on the other. Conflicts between park administrations and local communities (hereafter referred to as park–people conflicts) are some of the most pervasive problems, and can be extremely destructive [5,6].

In an attempt to reconcile conservation objectives with local livelihood interests and poverty alleviation, different strategies have been tested over the past decades to prevent and mitigate park–people conflicts in a peaceful manner. Among these, co-management has been one of the primary strategies employed by park administrations worldwide since the 1990s [5,7]. Here, we define it as participatory problem-solving arrangements in which the management of a territory or a set of natural resources is shared between a state administration and a community of resource users [7–9]. While initially co-management raised high hopes, today’s understanding is that it has not lived up to expectations [7,10] Widespread criticism has suggested that in practice co-management tends to gloss over the institutional complexities posed by the management of common-pool resources [7,11,12]. Co-management has also been criticized as a pretext to dissemble systems that empower the elite in the background while purporting to open up participation (e.g. [13]). In some cases, co-management has been blamed to actually exacerbate the conflict that it was intended to solve [5,9].

We hypothesize that part of the criticism on co-management results from the nature of classic evaluations. The majority of evaluation studies have only focused on conceptual assessments of whether and how local governance schemes, including co-management, contribute to the sustainable management of natural resources [14–17]. Systematic assessments of the role of specific enabling co-management conditions in conflict prevention at grassroots level are non-existent. Furthermore, co-management studies have traditionally approached state–community cooperation schemes as though each side were a homogenous entity composed of members who experience co-management (and conflict) in the same way. This ignores the fact that state authorities and communities are rarely coherent and homogeneous units [9,18,19]. Community members often have divergent and incommensurable perceptions of resource management and may experience co-management and conflict differently [20].

There is a pressing need for field assessments of the potential of co-management for lowering the occurrence of conflict [5]. In this paper we address this gap in research by focusing on the role of co-management in mitigating park-people conflicts in Colombia. Our goal was to assess whether the fulfilment of enabling co-management conditions as perceived by residents from ten Colombian national protected areas (NPAs) reduced their reported experience of park-people conflicts.

## Theoretical Context

### Conflict

Scholars differ in opinion on the best way to identify conflict and elucidate its underlying causes. Conflict has traditionally been defined as differences in goals, perceptions or interests between different (groups of) individuals [21]. According to this approach, differences should be appropriately addressed and reduced to reach effective conflict management. However, it is increasingly argued that this traditional view of conflict is not helpful for the development of lasting solutions to various natural resource-related conflicts because it does not distinguish conflict from its underlying causes [22]. Conceptualizing conflict as ‘differences’ distracts attention from the fact that non-conflict situations exist since ‘differences’ are inevitable in almost all social encounters.

We therefore applied the more recently developed impairment approach [22,23] which defines conflict as a situation in which an actor (here, local people) perceives ‘impairment’ through the action or behavior of another actor (here, generally the NPA administration as a ‘*pars pro toto*’, i.e. the state authorities it represents) due to their different perceptions, emotions and interests. According to this theory, at its core, conflict is a two-actor situation in which one actor, the “proponent”, acts to impair another actor, the “opponent”, with “actors” representing individuals or organizations [22]. One of the advantages of the impairment approach is that the experience of an actor’s behaviour/action as impairment is the only defining element used to distinguish conflict from non-conflict situations [22,23].

### Co-management

Understood as a system of joint decision making between state agencies and local communities, co-management allows all parties involved to negotiate, define and guarantee equitable sharing of management functions, entitlements and responsibilities for a given territory or set of natural resources [7,9,17]. Co-management thus distinguishes itself from other forms of participatory natural resource management though application of a philosophy of power-sharing and promoting partnerships [9]. Power-sharing arrangements can manifest themselves in many forms, and this variation is often depicted along a continuum [e.g. 24]. At one extreme are arrangements in which full control remains with the state agency, in spite of consultation with local communities. At the opposite end are arrangements in which local communities possess full control over the resources with little contribution from state agencies. Power-sharing arrangements at either end of this continuum, and everywhere in between, are here referred to as ‘co-management’.

It has hypothesised that an inverse relationship exists between sustainable resource management, including conflict resolution, and the fulfilment of a number of enabling co-management conditions, such as a sense of ownership over, or effective participation in, the co-management process by all individuals affected [17,24–27]. Our identification of enabling conditions that determine the potential for co-management to prevent or mitigate park–people conflicts is based on related research domains of common property theory and institutional analysis [24–28]. Within these theoretical frameworks, conflict resolution mechanisms are recognized as one of the key design principles for robust institutions, whereby institutions are understood as the conventions, values and formally sanctioned rules of a society that largely determine individuals’ perceptions of the world and their behavior in interacting with it [25]. Institutional analysts argue that all resource management models are embedded in broader institutional contexts and that (co)-management models can be understood as systems of governance, suggesting high relevance of institutional analysis for the management of park–people conflicts [25].

The literature on co-management has identified as many as 28 conditions that are critical for the sustainable governance of common pool resources [24–27]. Several of these are directly relevant for the contribution of co-management to conflict prevention and resolution. We selected ten conditions, which allowed using individual participants’ judgments as the sole criterion to assess the fulfilment of enabling co-management conditions (See Table 1 for full list of conditions and sub-conditions). For example, we included *effective participation*, but discarded *enabling policies and legislations* among the conditions. Our reasoning was that co-management participants can be expected to be able to self-asses or explain their involvement in making and changing co-management rules (i.e. a subcondition for ′effective participation′) whereas all respondents could not realistically be expected to be aware of, or able to comment on all relevant co-management-related legislations.

**Table 1:** Enabling co-management conditions and interpretation.

Co-management conditions	Co-management sub-conditions
**Individual incentive**	Participants feel that the co-management process benefits them and that they are better off complying than not complying with rules
	Alternatives are provided in case of access restriction
**Coordinating body**	There is a formal and operative body for co-management representing all stakeholder groups
**Trust**	Participants trust NPA functionaries
**Ownership**	Participants agree with the area of interest being a NPA and are willing to obey legislation and management rules
	In cases where there is some form of co-management, participants support it
**Effective participation**	Participants are involved in making and changing rules
	Local leaders are involved in making and changing rules
	There is at least one person of the local community appointed as park employee
**Free access to information**	Participants have free access to information (budgets, operational plans, etc.) when required
**Clear objectives**	Participants are aware of and understand basic NPA and co-management objectives, activities and scope. This includes for example whether or not people know if they live inside or outside the NPA, who else is involved in the co-management, etc.
**Empowerment**	There is capacity building related to NPA and co-management objectives and activities if relevant. This includes the socialization of the NPA Management Plan to the community.
**Compliance**	NPA Administration complies with prior informed consent procedures and/or with other (co-management) agreements and commitments
**Conflict management**	There exists a formal, identified, consensual and functioning conflict management mechanism at the local level, where solutions to conflicts can be quickly resolved
	There is regular and informal communication between both parties about how conflicts can be resolved

## Materials and Methods

### Research areas and background

**Fig 1:**
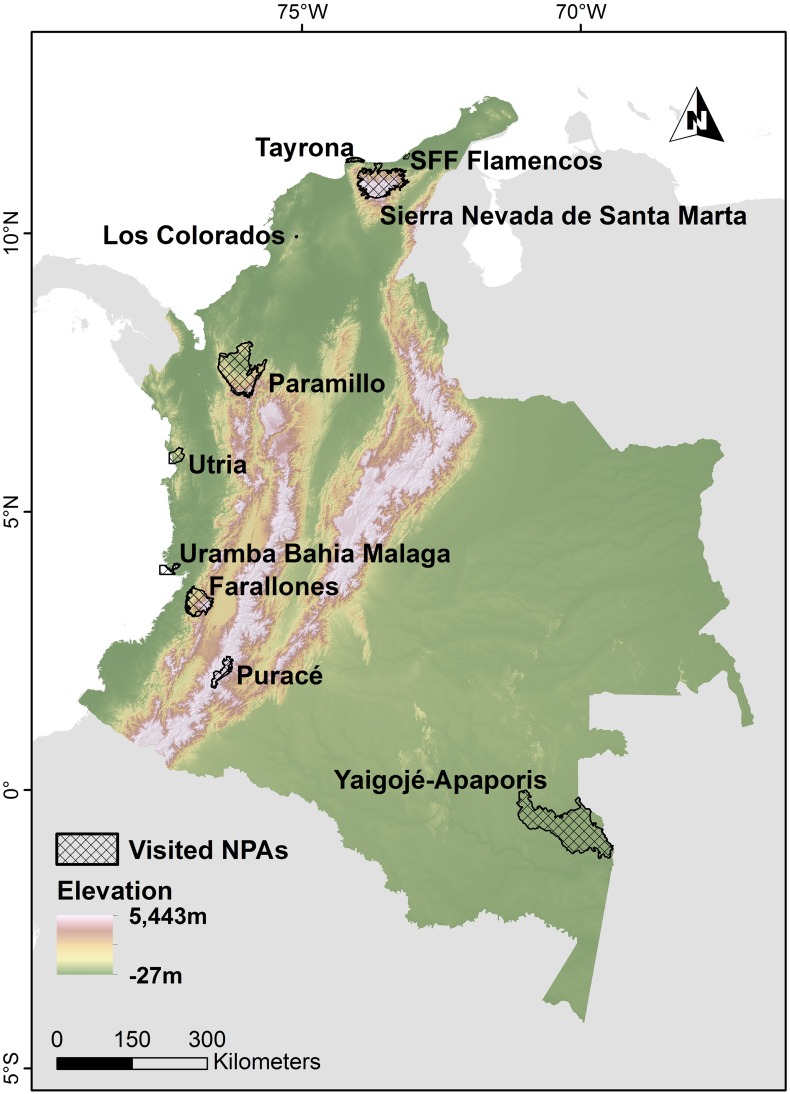
Location of the visited NPAs (for more details please see Table 2).

Between 1 October 2011 and 31 August 2014, we carried out fieldwork in ten Colombian NPAs (Fig 1), which are located in the main Colombian bio-cultural regions (Amazon, Andes, Caribbean and Pacific coasts) and are home to various indigenous, Afro-Colombian and settler communities. These NPAs covered surface areas from 1,000 to 1,000,000 hectares; some were created decades ago while others came into existence more recently. Due to the complexities of the NPAs (in terms of size, high numbers of ethnic groups, the presence of armed groups, difficulties of access, etc.) we focused on specific subareas defined on the basis of socio-cultural and geographical coherence. For some NPAs we selected more than one subarea (Table 2).

**Table 2:** Characteristics of the NPAs and study areas considered in this paper.

NPA	Region	Year of NPA creation	Surface area (ha)	Areas of residence of respondents (N = 2584)	Co-management in the selected study areas
SFF Los Flamencos	Caribbean	1977	7,615	Cari Cari and Palaima (n = 8)	No
				Indigenous Wayuu collective territory “Perratpu” (case Flamencos) (n = 43)	Yes
				Displaced community near Tocoromana (n = 9)	No
				Afro-Colombian communities Los Cocos and Camarones (n = 7)	No study area
Tayrona				• Settler/fisher communities Tayrona (case Tayrona) (n = 61)	• No
	Caribbean	1964	15,000	Indigenous community Tayrona (n = 4)	No study area
Sierra Nevada de Santa Marta	Caribbean	1964	383,000	Settler communities La Lenguëta (case Sierra Nevada) (n = 60)	No
				Indigenous collective territory Kogui-Malayo-Arhuaco (n = 10)	No
				Indigenous community Kankuamo (n = 1)	No study area
SFF Los Colorados	Caribbean	1977	1,000	Settler communities Los Colorados (case Colorados) (n = 38)	No
Utria	Pacific	1987	54,300	Afro-Colombian community councils (case Utria Afro-Colombian) (n = 66)	Yes
				Indigenous collective territory “Jurubida-Chori-Alto Baudo” (case Utria indigenous) (n = 41)	Yes
				Indigenous collective territory Alto Rio Valle Boro Boro (n = 1)	No study area
Los Farallones	Pacific	1968	205,266	Afro-Colombian community councils Los Farallones (n = 8)	Yes
Uramba Bahia Malaga	Pacific	2010	47,094	Afro-Colombian community councils Bahia Malaga (case Bahia Malaga) (n = 74)	Yes
Paramillo	Andes	1977	460,000	Indigenous collective territory “Yaberarado” (case Paramillo) (n = 20)	Yes
				Indigenous collective territory “Pollines” (n = 2)	No study area
Puracé	Andes	1975	83,000	Indigenous collective territory Puracé (case Puracé)	No
				Indigenous collective territory Rio Blanco (n = 2)	No study area
				Settler community Puracé (n = 2)	No study area
Yaigojé-Apaporis	Amazon	2009	1,056,023	Indigenous collective territory “Yaigojé-Apaporis” (case Yaigojé-Apaporis) (n = 85)	Yes

In response to conservation failures and escalating park–people conflicts, the Colombian Ministry of Environment adopted a new conservation approach in 2001, which abandons the absolutist conservationist stance (i.e. parks without people) and focuses instead on dialogue and participation, drawing upon co-management and participatory conservation models [29]. Until now there have been no attempts to assess the effectiveness of these models. Overall, our study cases represent a variety of park-people conflicts, against a background of variable degrees of co-management (Table 2).

### Data collection and analysis

We interviewed 584 representatives of various resident indigenous, Afro-Colombian and newcomer groups, such as small-scale farmers and fisher communities [30]. Nineteen of them did not actually live in the subareas mentioned above, but were nonetheless residents of the visited parks (Table 2). Interviews were conducted during several field trips, mostly in the local settlements (e.g. in respondents’ houses or public places). Committed to abide to ethical standards, we always tried to protect participants’ rights and privacy as much as possible. The scholarship for this research was approved without the need for evaluation of an ethics committee or IRB because of the nature of the interviews. The purpose of the study was not to observe, study or test individuals but rather to collect aggregated data for the evaluation of our research hypotheses. Also, we did not try to obtain access to traditional knowledge, or to other types of knowledge protected by international or national regulations and legislation. We only asked questions to people about their individual experience of conflict with NPA administrations, and their perceptions of the fulfilment of enabling conditions considered necessary for exogenously imposed co-management schemes to work (see S1 Table for list of interview questions). The participating communities and Colombian NPA administration fully authorized this working method and the latter formalized its agreement with this research in a written document.

When arriving to each of the participating communities, in a first step permission was obtained from organized community assemblies, during which we thoroughly explained the goals and limitations of the research. Based on this, the communities collectively decided if, and under what conditions we were allowed to carry out interviews. In a next step we sought every individual respondent’s verbal informed consent before proceeding with the interview. For participants under the age of 18, consent was sought and obtained from their parents and/or guardians. Consent was recorded in writing by the interviewer. We always interviewed community leaders first (i.e. presidents of the community councils, teachers, traditional leaders, etc.) to obtain a general overview of the local context. Next, we interviewed individual community members making sure to select a representative sample of different interest groups. All conversations were recorded in writing during interview sessions. Audio recordings were not used and data was treated anonymously and confidentially.

During interviews we collected data on: (i) a set of personal attributes (sex, age, income and education level, ethnic background, residence status, position in the community, language proficiency, economic and productive activities, work relation with NPA administration, and land occupation and tenure); (ii) experience of park–people conflicts; and (iii) perception of the fulfilment of enabling co-management conditions (see S1 Table for a list of interview questions).

Our approach to analyse conflict and the capacity of co-management to prevent or mitigate it builds on the widely shared and applied Thomas Theorem—“*if men [sic] define situations as real*, *they are real in their consequences*” [31], thus accommodating the fact that people’s perceptions are decisive for their experience of conflict. Moreover, people also *act* upon their perceptions, according to this theory, thus (re)producing more or less intense conflict situations. To capture such perceptions, research methods should pay thorough attention to people’s subjective meanings and experiences. To assess individual respondents’ experiences of park-people conflicts, we explicitly asked them to free-list all types of actions they perceived as impairment. All interviews were carried out by the first author; hence potential interviewer bias can be expected to be constant across the cases.

Collinearity of enabling conditions has been raised as an issue in co-management assessments [16,32]. However, neither the set of enabling conditions, nor the conflict categories we selected showed strong signs of collinearity (variance inflation factors smaller than 10 and 2, respectively), justifying their inclusion [33]. We applied an iterative modelling approach based on Aikake’s information criteria (AIC) [34]—employing generalized linear models with a binomial distribution and logit link function—to identify potential explanatory variables that significantly contributed to explaining the experience of conflict by respondents and, hence, to evaluate the power of co-management to prevent or mitigate conflict. The response variable was the proportion of most-important conflicts reported to be experienced by individual respondents. Our initial set of explanatory variables was a combination of the perceived degree of fulfilment of the different enabling conditions by respondents and the set of personal attributes mentioned above (i.e. sex, age, etc.). We also examined pairwise relationships between the fulfilment of different co-management conditions and the reported experience of different park–people conflicts by means of logistic generalized linear models.

## Results and Discussion

All conflict situations mentioned by the respondents were organised across nine categories, of which we will focus on the five most important ones, being those reported by more than one third of all respondents, i.e. constrained socio-economic development, access restriction, non-compliance, constrained communication and participation, and imposition of exogenous objectives (see Table 3 for full definitions).

**Table 3:** Five most important conflict categories reported by respondents (N = 584).

**Constrained socio-economic development (reported by 50% of respondents)** covers three types of conflicts. First, it refers to a restriction of infrastructure development enforced by the NPA administration (mainly house building projects). Second, it includes the impediment of certain local development projects (e.g. the building of a school, community-based tourism activities, road construction, the provision of public services such as natural gas or electricity); and thirdly, it refers to limited or non-existent sharing of benefits resulting from NPA-governed activities (e.g. income from tourism). **Access restriction (48%)** denotes actions intended to prevent people from having access to a particular resource or area while often at the same time ensuring the imposer’s own access to it. Access restrictions vary in scale and intensity, and can be summarized as: (i) restriction on extraction and/or use of natural resources (e.g. timber extraction, fishing and hunting, cattle breeding, agricultural practices, etc.); (ii) restriction on access to land and/or entrance to territory (e.g. people are not allowed to stay overnight within the NPA as they did before, or have to pay entrance fees; children are not permitted to enter the NPA); and (iii) restriction on land tenure regimes and rights, including the obstruction of the legalization or formalization of land ownership and prohibition of selling and buying of land. **Non-compliance (47%)** with agreements or previously defined rules can also lead to conflict. NPA administration is often accused of not complying with: (i) prior-informed consent procedures (PICP; e.g. related with the appointment of park functionaries or the approval of scientific research projects); (ii) (co-management) agreements and promises to adequately reflect community interests in NPA management plans and development projects; and (iii) promises to support community-based organizations. Some community members also complained about the cancellation of meetings by NPA officials without clear justification. We have grouped all these conflicts together as respondents generally did not distinguish between non-compliance with PICP and non-compliance with (co-management) arrangements. **Constrained communication and participation (42%)** refers to actions that intentionally or unintentionally limit participation of stakeholders in NPA and co-management decision-making. We distinguish between four dimensions of constrained participation: (i) constrained or restricted local leadership in NPA management and administration; (ii) a limited number of local park employees; (iii) constrained or limited coordination and communication between NPA staff and local communities; and (iv) barriers to community access to information (or non-availability in the appropriate language): e.g. no or limited access to NPA budget, profit and expense figures; limited or non-existent information flow about the location of NPA borders, NPA objectives, the environmental legislative framework, authorized and forbidden activities, ongoing research (projects), agreements signed between NPA administration and local leaders, etc. Furthermore, in some cases there seemed to be a lack of environmental education. Respondents complained that they were being forbidden certain activities without knowing or understanding the environmental motives underlying these restrictions. **Imposition of exogenous objectives (38%)** refers to actions undertaken to implement or pursue management objectives or goals of a particular stakeholder group (i.e. NPA administration) beyond the will or interests of other groups (i.e. the community). For instance, NPA Administration was accused by some of enforcing the imposition of NPAs on ancestral lands, and of the forced removal or resettlement of communities outside NPAs.

The most parsimonious combination of variables that explained people’s experience of conflict were: (i) the area where a person resided; (ii) trust in NPA staff and (iii) the feeling that they could effectively participate in the co-management process (Table 4). This means that it is the simplest plausible subset of all the variables we measured that best explain the variation in the numbers of conflict categories reported by the people we interviewed. It does not mean that the other variables are necessarily insignificant, as discussed below.

**Table 4:** The most parsimonious set of variables retained by iterative modeling approach based on generalized linear model with a binomial distribution and logit link function (only study areas with at least 8 respondents; N = 565). The first line reports on residual deviance and AIC of the model. In the next lines, values for these parameters are given for the case in which each individual variable is removed from the model, together with the significance of the difference. This model explains 59% of the null deviance.

Explanatory variable	Df	Deviance	AIC	LRT	Pr (>Chi)
		*100*.*25*	*395*.*14*		
Area where a person resides	13	*159*.*27*	*428*.*16*	*59*.*019*	*7*.*7e-08*
Effective participation condition	1	*112*.*46*	*405*.*34*	*12*.*203*	*4*.*8e-04*
Trust condition	1	*111*.*76*	*404*.*64*	*11*.*504*	*6*.*9e-04*

**Fig 2:**
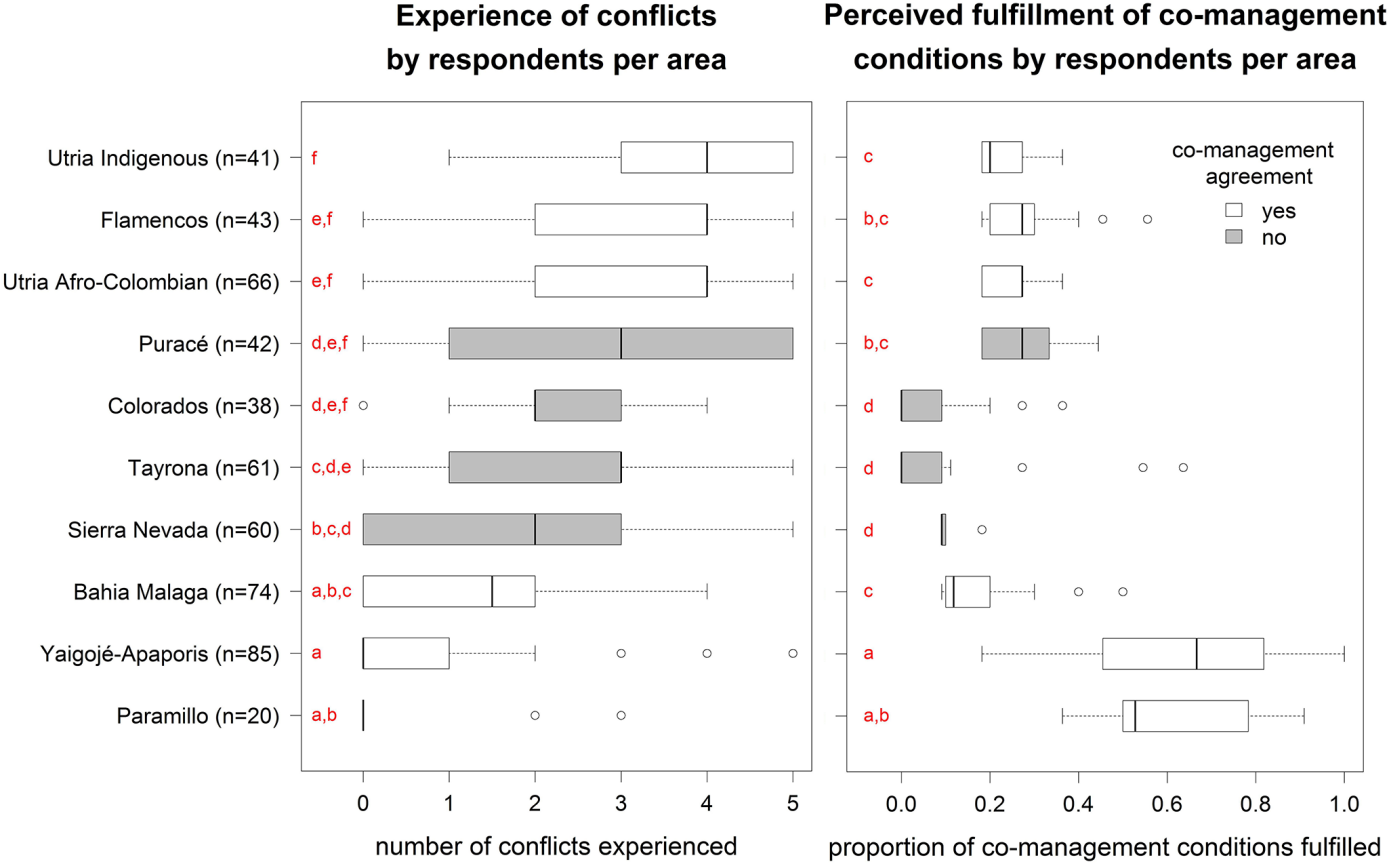
Distribution of the experience of conflict and the perception of the fulfillment of co-management conditions according to residents of the different study areas where n≥20 (N = 530). Distributions were significantly different (*P*<<0.001) across study areas (Kruskall-Wallis chi-squared = 177.04 and 352.39 for conflict and co-management conditions, respectively). Letters indicate groups of study areas with similar distributions, based on multiple comparison post-hoc tests (threshold at *P* 0.01)[35].

The importance of a person’s residence was not related to the existence of a formal co-management agreement between local communities and NPA administration. In four of the six study areas (n≥20) where co-management agreements had been signed, respondents reported a similar number of conflict as in the four areas where such agreements were lacking (Fig 2). The fulfilment, as perceived by respondents, of co-management conditions was a more important predictor of conflict than the actual existence of a written co-management agreement. Only in those study areas where co-management agreements had been signed and where more than half of the respondents considered more than half of the enabling conditions to be fulfilled, were individual experiences of conflict close to zero (Fig 2). It is interesting to note that while for most study areas there was quite some variation in the number of conflict conditions reported by the respondents, there seemed to be more agreement on the proportion of co-management conditions perceived to be fulfilled, as evidenced by the wide and narrow nature of boxplots on the left and right panels of Fig 2, respectively. These findings were anticipated since conflict is influenced by many factors, including personal ones, whereas the fulfillment of co-management conditions can be expected more a matter of external factors, and hence less susceptible to high variation between individuals. If this interpretation is correct, the results suggest that the median values of proportions reported by respondents per area (Fig 2 right panel), are likely to be realistic approximations of reality. Only for the two areas where respondents said more than half of the co-management conditions to be fulfilled (i.e. Paramillo and Yaigojé-Apaporis), perceptions of the numbers of fulfilled co-management conditions were more variable, the reason for which is less clear to us.

Interestingly, our most parsimonious model solution suggests that co-management conditions were more decisive determinants of people’s reported experiences of conflict than social diversity characteristics such as sex, ethnic background or level of education (Table 4). From a policy perspective, this is encouraging, as state authorities might find it more straightforward to influence the fulfilment of enabling conditions than to influence most social diversity attributes, at least in the short term.

**Fig 3:**
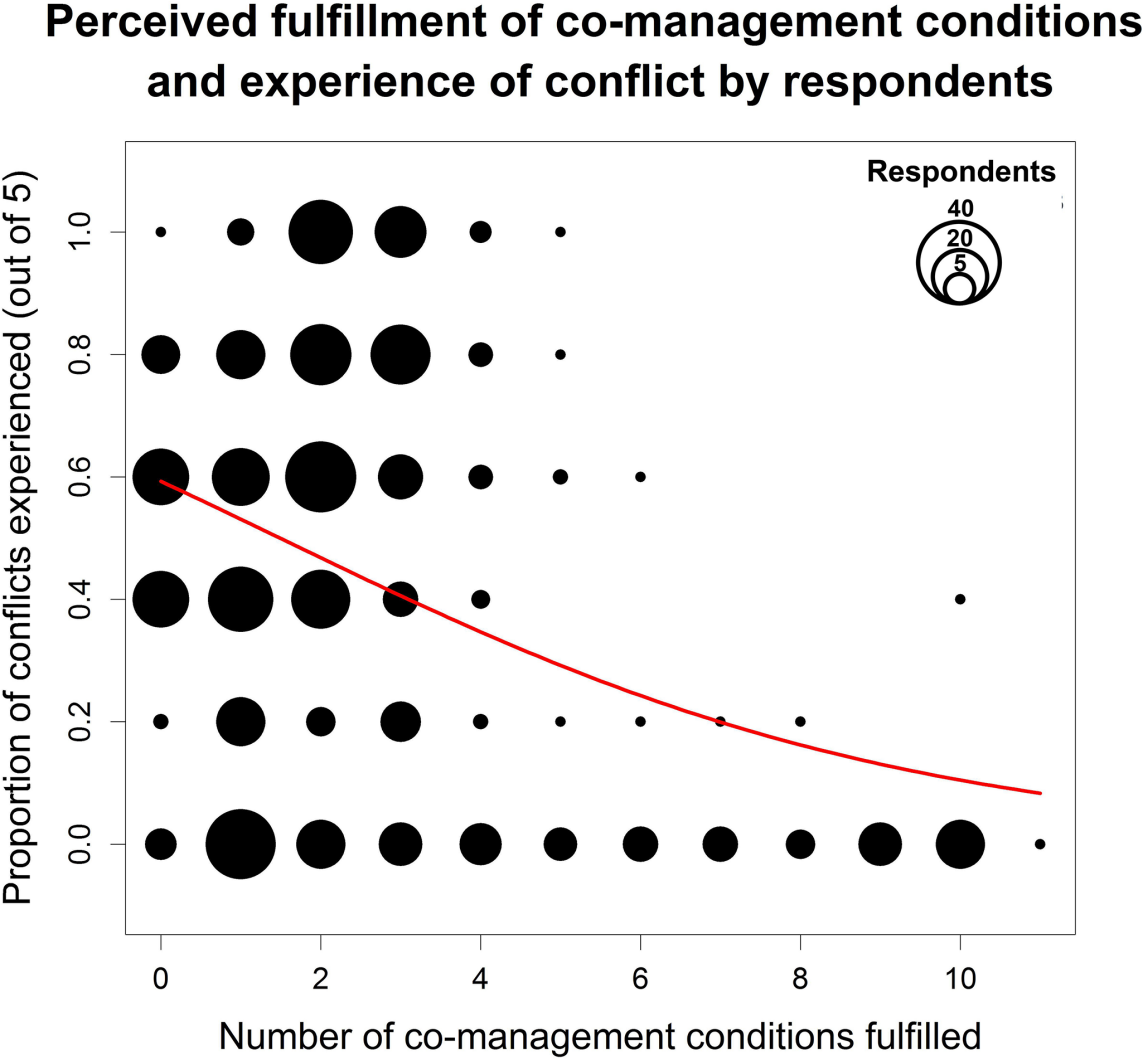
Conflicts reported to be experienced by people as a function of the number of co-management conditions perceived to be fulfilled (GLM with binomial distribution and logit link function; N = 584; z = -5.68; *P* = 1.3e-8). A condition was considered fulfilled if at least one of its sub-conditions was met (see Table 1 for list of sub-conditions). This model explains 11% of the null deviance.

Although proportionally few people considered more than five enabling co-management conditions to be met (11%), we found a strong inverse relationship between the fulfilment of enabling conditions and the perception of conflict by respondents. Fig 3 shows that the vast majority of people who considered at least four conditions to be met (85 out of 114; 75%) did not experience any conflict.

**Fig 4:**
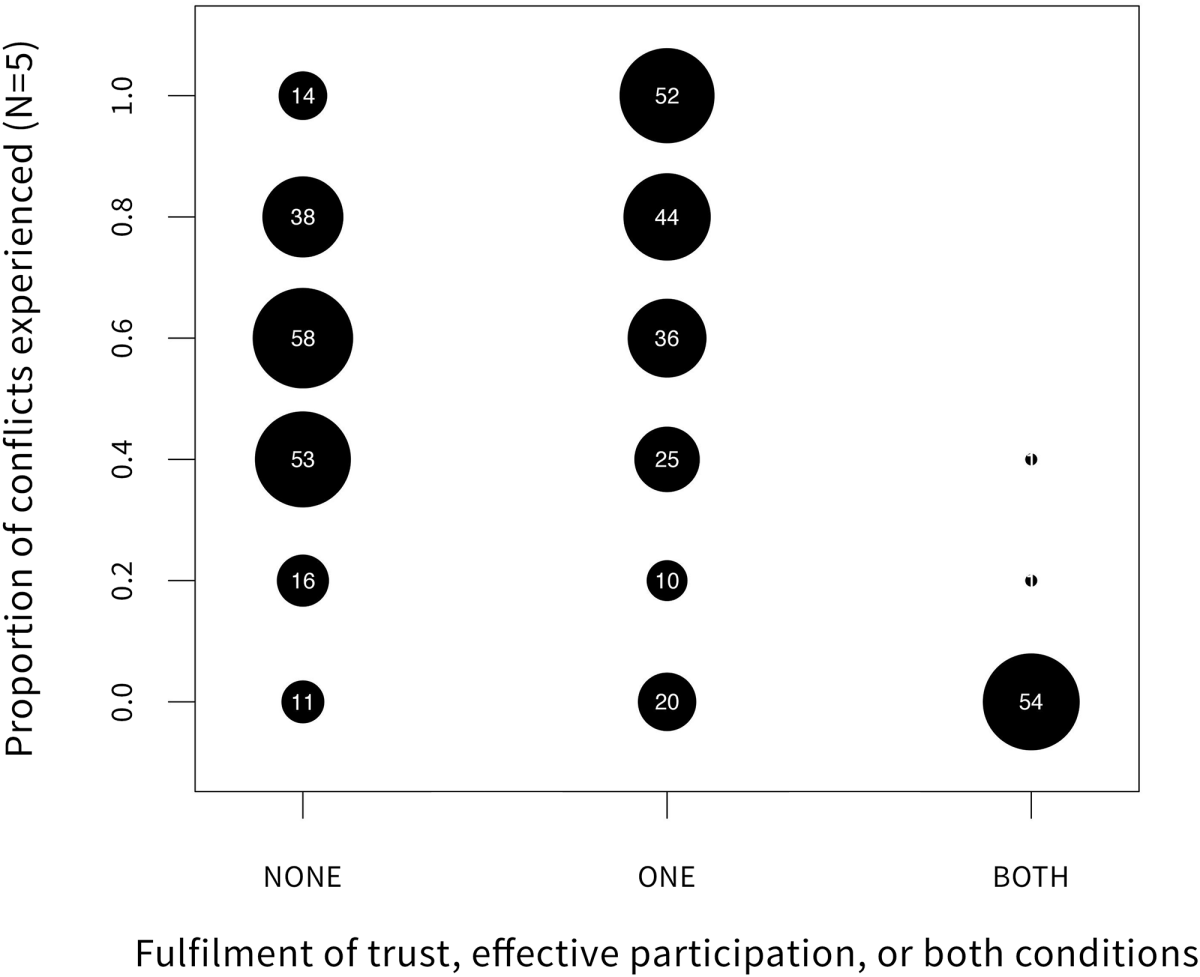
Distribution of the reported experience of conflict and the perception of the fulfilment of trust and effective participation conditions according to residents of the different study areas.

In line with the variables retained by our model (Table 4), the most effective combination of co-management conditions was trust in NPA staff and effective participation: 96% of respondents who considered these conditions fulfilled reported no experience of conflict (Fig 4). This finding suggests that trust-building and ensuring the effective participation are the most decisive factors among the extensive list of conditions for lowering conflict listed in Table 1. Hence, they should be priority tasks for governments and NPA administrations interested in preventing or mitigating park-people conflicts on a tight budget.

The importance of trust and effective participation for participatory environmental management is well-documented in literature. Environmentalists, policy practitioners and research scholars have repeatedly highlighted the vital role of participation and public involvement in a range of conservation development concerns, including implementing policies for environmental protection, and co-management [25,36–38]. Participation can improve learning processes and the quality of decision-making. It encourages empowerment, democratic citizenship and public support for planning decisions. As a consequence, it can lead to effective and efficient implementation processes as well as the prevention or mitigation of conflicts [38,39]. Likewise, trust has been identified as a highly influential factor in the success or failure of environmental management and cooperation agreements [27,28,40,41]. Trust is a key requisite for peace building within participatory resource management processes [27,28,40] and a vast literature exists about the factors that make the greatest contribution towards building trust between individuals, groups and the organizations and societies to which they belong (e.g. [25, 40–42]).

An evaluation of pairwise relations between the reported fulfilment of conditions and experiences of conflict revealed that, although the level of fulfilment of most conditions reported by respondents was generally low, all but two co-management conditions correlated negatively with at least one conflict category. Table 5 suggests that, in addition to (i) trust-building between local people and NPA staff, and (ii) ensuring local people’s effective participation in the co-management process, priority conditions in which governments and park administrations should invest are: (iii) stimulate ownership over the co-management process, (iv) operationalize a coordinating body and (v) create individual incentives, all of which significantly lowered the experience of at least four out of five conflict categories.

**Table 5:** Pairwise relations between idiosyncratic perceptions of the fulfillment of co-management conditions and the reported experience of conflict categories, based on GLMs with binomial distribution and logit link function. Numbers correspond to z and *P* values; significant at *P*<0.01 are in bold. All significant relations refer to inverse correlations. Percentages in row and column heads refer to the number of respondents experiencing different conflict categories and considering a specific enabling condition fulfilled. For enabling conditions with sub-conditions (see Table 1), degree of fulfillment was calculated as the proportion of all sub-conditions queried.

	Conflict categories	Constrained development (52%)	Non-compliance (48%)	Access restriction (49%)	Constrained participation (43%)	Imposition objectives (38%)	Total number of conflicts experienced
Co-management conditions							
**Individual incentive (10%)**	Z =	-4.13	-3.25	-3.89	-3.52	-3.30	-4.34
	P =	**3.6e-05**	**1.14e-03**	**1.02e-04**	**4.31e-04**	**9.71e-04**	**1.41e-05**
**Coordinating body (8%)**	Z =	-4.1	-3.88	-3.35	-3.5	-2.88	-4.66
	P =	**4.09e-05**	**1.05e-04**	**8.17e-04**	**4.59e-04**	**4.02e-03**	**3.11e-06**
**Trust (6%)**	Z =	-4.23	-4.15	-3.07	-3.07	-3.12	-4.51
	P =	**2.32e-05**	**3.33e-05**	**2.12e-03**	**2.17e-03**	**1.79e-03**	**6.61e-06**
**Ownership (16%)**	Z =	-5.48	-4.11	-5.16	-3.31	-2.07	-4.87
	P =	**4.27e-08**	**4e-05**	**2.53e-07**	**9.22e-04**	3.85e-02	**1.12e-06**
**Effective participation (20%)**	Z =	-4.41	-4.46	-3.59	-0.31	-1.42	-3.78
	P =	**1.04e-05**	**8.14e-06**	**3.27e-04**	7.57e-01	1.56e-01	**1.59e-04**
**Free access to information (3%)**	Z =	-2.64	-2.58	-2.02	-1.84	-1.88	-2.85
	P =	**8.38e-03**	**9.76e-03**	4.32e-02	6.55e-02	6.01e-02	**4.44e-03**
**Clear objectives (5%)**	Z =	-3.43	-1.43	-2.75	-1.86	-0.91	-2.36
	P =	**6.07e-04**	1.52e-01	**6e-03**	6.35e-02	3.64e-01	1.84e-02
**Empowerment (7%)**	Z =	-3.37	-1.5	-2.06	0.43	-2.37	-2.35
	P =	**7.45e-04**	1.32e-01	3.92e-02	6.66e-01	1.76e-02	1.89e-02
**Compliance (4%)**	Z =	-0.02	-0.02	-0.02	-2.37	-0.02	-2.29
	P =	9.84e-01	9.84e-01	9.84e-01	1.77e-02	9.85e-01	2.22e-02
**Conflict management (3%)**	Z =	-1.69	-9.74e-01	-1.65	-8.66e-01	-6.16e-01	-1.35
	P =	9.04e-02	3.3e-01	9.98e-02	3.86e-01	5.38e-01	1.77e-01

## Conclusion

In recent years, co-management has lost much of its appeal as an effective mechanism for preventing or resolving natural resource-related conflicts worldwide. In contrast to this understanding, our findings reveal -for the first time quantitatively- that it is premature to discard co-management as a tool for conflict resolution. We call upon park administrations and policymakers to invest in ensuring the fulfilment of enabling conditions for co-management not only on paper, but also, and more importantly, by praxis. Our findings suggest that building trust between partners and achieving more effective participation of local groups in NPA management are most-important conditions to prevent or mitigate park-people conflicts, which can be a daunting task, but surely far from impossible. Dismissing the potential effectiveness of co-management is likely to result in the status quo of conflicts, or lead to their escalation, with potentially detrimental consequences for the conservation of biodiversity. This is particularly relevant for countries such as Colombia, which are in post-conflict decision-making processes at numerous levels.

Looking ahead, further work is necessary to determine to what extent the outcomes of this study can be generalized to co-management schemes in protected areas and other common property contexts in the global South, such as in fisheries or forestry. Also, indicators to monitor the state and progress of the fulfilment of enabling conditions will need to be developed and measured. Furthermore, a number of other outstanding research questions remain. More work is necessary to improve our understanding of the extent to which the number of conflicts experienced by respondents reflect their actual manifestation or escalation on the ground. History has taught that certain tipping points must be crossed for conflict to escalate, but it is not clear if this also applies to park-people conflicts, and if so, how to ensure conflict to stay within constructive limits (e.g. [43]). Lastly, more research is necessary to better understand if and how the fulfilment of co-management conditions on the ground actually also results in improved biodiversity conservation in protected areas.

## Supporting Information

S1 Table List of interview questions. (DOCX)
