# Supplementary material for: Conflict in Protected Areas: Who Says Co-Management Does Not Work?
Source: PLoS One. 2015 Dec 29;10(12):e0144943. doi: 10.1371/journal.pone.0144943 (PMC4695097; doi:10.1371/journal.pone.0144943)
Supplement: S1 Table — (DOCX) [file pone.0144943.s001.docx]

**Supporting Information**

**Table S1. Interview list**

1. What is your name?
2. Are you male/female?
3. How would do you define your ethnic background?
4. What languages do you speak?
5. How old are you?
6. What is your education level (no education, primary school, secondary school, subsequent studies)
7. What is your income level (no salary, less than min salary, min salary, more than min salary)?
8. What is your position in the community (ancestral leader, teacher, community member….)?
9. Is the community in which you live located within/along side a protected area?

- If 9 is yes: 9.1. What is the name of this protected area? 9.2. What are the main formal objectives of the protected area?

1. Are you the owner of the land you occupy? Do you possess any land property acts? Please explain.
2. Do you engage in economic/productive activities?

- If so: 11.1. Do you undertake these activities within or outside of the NPA?

1. Have you ever been employed by the NPA administration? Please explain.
2. Have you experienced the NPA administration as an obstacle or a threat to your well- being?

- If so: 13.1. Can you explain this in detail? In what way do you feel the NPA administration is a threat to you well-being?

1. Do you have free access to information regarding the NPA (budgets, operational plans, etc.) when requested? Please explain.
2. Do you feel involved in making and changing rules regarding NPA management? Please explain.
3. How many members of your community are appointed as park employees?
4. Do you feel well informed about the decisions and activities taken by the NPA administration that affect you,? Please explain.
5. Do you trust NPA functionaries? Why or why not?
6. Do you know of any agreements (that are/were implemented) between the NPA administration and your community?

- If so: 19.1. When were they signed?

19.2. What are/were these agreements about?

19.3. What are/were the basic objectives of these agreements?

19.4. Do you feel these agreements generate/have generated benefits for you? Please explain.

19.5. Do you support these agreements? Why or why not?

19.6. Do you feel you are better off complying than pushing against these agreements? Please explain.

19.7. Do these agreements encompass any kind of restriction of access to natural resources?

19.7.1. If this is the case, are alternatives provided to cover this loss? Please explain.

19.8. Is there a formal representative body designated to follow up on these agreements?

19.8.1.If this is the case, is this body functional?

1. Do you agree or disagree that this area should be a NPA? Please explain.
2. Are you willing to obey NPA legislation and management rules? Please explain.
3. Have you received capacity building (for example explanations, working classes, etc.) related to NPA and co-management objectives and activities? Please explain.
4. Was the NPA management plan explained and socialized to the community? Please explain.
5. Does the NPA administration comply with previously determined consent procedures and/or with (co-management) agreements and commitments? Please explain.
6. Is there a formal, identified, agreed upon and functioning conflict mechanism for solving issues at the local level? Please explain.
7. Is there regular and informal communication between the community and NPA administration about how conflicts can be resolved? Please explain.
